# Whole genome sequencing reveals a frameshift mutation and a large deletion in YY1AP1 in a girl with a panvascular artery disease

**DOI:** 10.1186/s40246-021-00328-1

**Published:** 2021-05-10

**Authors:** Víctor Raggio, Nicolas Dell’Oca, Camila Simoes, Alejandra Tapié, Conrado Medici, Gonzalo Costa, Soledad Rodriguez, Gonzalo Greif, Estefania Garrone, María Laura Rovella, Virgina Gonzalez, Margarita Halty, Gabriel González, Jong-Yeon Shin, Sang-Yoon Shin, Changhoon Kim, Jeong-Sun Seo, Martin Graña, Hugo Naya, Lucia Spangenberg

**Affiliations:** 1 Departamento de Genética, Facultad de Medicina, Universidad de la República, General Flores 2125, 11800 Montevideo, Uruguay; 2 Bioinformatics Unit, Institut Pasteur de Montevideo, Mataojo 2020, 11400 Montevideo, Uruguay; 3 Cátedra de Neuropediatría, Centro Hospitalario Pereira Rossell, Facultad de Medicina, Universidad de la República, General Flores, 2125 Montevideo, Uruguay; 4 Molecular Biology Unit, Institut Pasteur de Montevideo, Mataojo, 2020 Montevideo, Uruguay; 5 Departamento de Pediatría, Centro Hospitalario Pereira Rossell, Facultad de Medicina, Universidad de la República, General Flores 2125, 11800 Montevideo, Uruguay; 6 Departamento de Pediatría, Nefrología pediátrica, Centro Hospitalario Pereira Rossell, Facultad de Medicina, Universidad de la República, General Flores 2125, 11800 Montevideo, Uruguay; 7 Precision Medicine Institute, Macrogen Inc., Seoul, 08511 South Korea; 8 Bioinformatics Institute, Macrogen Inc., Seoul, 08511 South Korea; 9 Precision Medicine Center, Seoul National University Bundang Hospital, Seongnam, South Korea; 10 Departamento de Producción Animal y Pasturas, Facultad de Agronomía, Universidad de la República, Av Gral Eugenio Garzón 780, 12900 Montevideo, Uruguay; 11 Department of Informatics and Computer Science, Universidad Católica del Uruguay, 8 de Octubre 2738, 11600 Montevideo, Uruguay

**Keywords:** Medical genomics, Bioinformatics, Neurology, Whole genome sequencing

## Abstract

**Background:**

Rare diseases are pathologies that affect less than 1 in 2000 people. They are difficult to diagnose due to their low frequency and their often highly heterogeneous symptoms. Rare diseases have in general a high impact on the quality of life and life expectancy of patients, which are in general children or young people. The advent of high-throughput sequencing techniques has improved diagnosis in several different areas, from pediatrics, achieving a diagnostic rate of 41% with whole genome sequencing (WGS) and 36% with whole exome sequencing, to neurology, achieving a diagnostic rate between 47 and 48.5% with WGS. This evidence has encouraged our group to pursue a molecular diagnosis using WGS for this and several other patients with rare diseases.

**Results:**

We used whole genome sequencing to achieve a molecular diagnosis of a 7-year-old girl with a severe panvascular artery disease that remained for several years undiagnosed. We found a frameshift variant in one copy and a large deletion involving two exons in the other copy of a gene called YY1AP1. This gene is related to Grange syndrome, a recessive rare disease, whose symptoms include stenosis or occlusion of multiple arteries, congenital heart defects, brachydactyly, syndactyly, bone fragility, and learning disabilities. Bioinformatic analyses propose these mutations as the most likely cause of the disease, according to its frequency, in silico predictors, conservation analyses, and effect on the protein product. Additionally, we confirmed one mutation in each parent, supporting a compound heterozygous status in the child.

**Conclusions:**

In general, we think that this finding can contribute to the use of whole genome sequencing as a diagnosis tool of rare diseases, and in particular, it can enhance the set of known mutations associated with different diseases.

**Supplementary Information:**

The online version contains supplementary material available at 10.1186/s40246-021-00328-1.

## Background

Rare diseases (RD) are pathologies that affect less than 1 in 2000 people [1]. They are difficult to diagnose due to their low frequency and their often highly heterogeneous symptoms. RDs have in general a high impact on the quality of life and life expectancy of patients, which are in general children or young people. Patients with RD must frequently overcome a “diagnostic odyssey,” in which they jump from specialist to specialist for long periods of time (in average 5 years) to get a proper diagnosis. This delayed diagnosis can have a big impact on the quality of life. Having a proper diagnosis is the first step towards getting proper medical care. In this context, medical genomics has been a powerful tool to help in the diagnosis of RDs. The advent of NGS techniques has improved diagnosis in several different areas, from pediatrics, achieving a diagnostic rate of 41% with whole genome sequencing (WGS) and 36% with whole exome sequencing (WES) [2], to neurology, achieving a diagnostic rate between 47 and 48.5% with WGS [3, 4]. In general, the use of WGS in RDs has had a diagnostic success of 62.5% in, for example, a Chinese study [5]. This evidence has encouraged our group to pursue a molecular diagnosis using WGS for this and several other patients.

Grange syndrome is a recessive disease that was first reported in 1998 in a family in which 4 of 9 siblings had a unique syndrome of stenosis or occlusion of multiple arteries, including renal, abdominal, cerebral, and probably coronary arteries; congenital heart defects; brachydactyly; syndactyly; bone fragility; and learning disabilities [6]. The arterial occlusive features were similar to fibromuscular dysplasia and the bone fragility aspect of the disease resembled a mild form of osteogenesis imperfecta. Several additional cases were reported in the following years [7, 8]. The diagnosis was difficult due to the heterogeneity of symptoms and resemblance to other conditions. In all those cases, homozygous or compound heterozygous mutations were found in YY1AP1. This gene localizes to the nucleus and encodes yin yang 1 (YY1)-associated protein 1; YY1AP1 and YY1 are components of the INO80 chromatin remodeling complex, which is responsible for transcriptional regulation, DNA repair, and replication. Studies in vascular smooth muscle cells showed that loss of YY1AP1 results in cell cycle arrest (specifically, G2 cell cycle arrest without evidence of apoptosis), with decreased proliferation and increased levels of the cell cycle regulator CDKN1A, and disruption of TGF-beta-driven differentiation of smooth muscle cells [9]. These alterations may substantially impact vascular smooth muscle cells, indeed the most compromised cell types in this pathology.

In this study, we report the case of a 7-year-old girl with a severe panvascular artery disease without a diagnosis. We performed WGS and found a frameshift variant in one allele and a large deletion in the other copy of the YY1AP1 gene. Given the evidence of in silico predictors, family segregation, conservation analysis, and population-based data, we believe we have enough evidence to support these variants’ pathogenicity.

## Results

### Case report

Here, we present the case of a 13-year-old girl born from healthy, non-consanguineous parents. She is the only daughter of the couple. Father has a healthy daughter from another previous couple. No other family history to highlight. Her mother’s first pregnancy was complicated with oligohydramnios and intrauterine growth restriction probably related to antiphospholipid syndrome. The patient was born at 36 weeks of gestation without relevant perinatal problems, with normal postnatal growth and development.

At the age of 6 years, hypertension and dyslipidemia (total cholesterol 284 mg/dl, low-density lipoprotein cholesterol (LDL-c) 205 mg/dl, high-density lipoprotein cholesterol (HDL) 59 mg/dl, triglycerides 106 mg/dl) were diagnosed, and subclinical hypothyroidism was detected and treated.

A few months later, she suffered two episodes of transient ischemic attack (TIA). Cranial tomography was normal, and no alterations were detected in neck vasculature by ultrasound at that time. Magnetic resonance imaging was not performed at the time for unknown reasons.

**Fig. 1:**
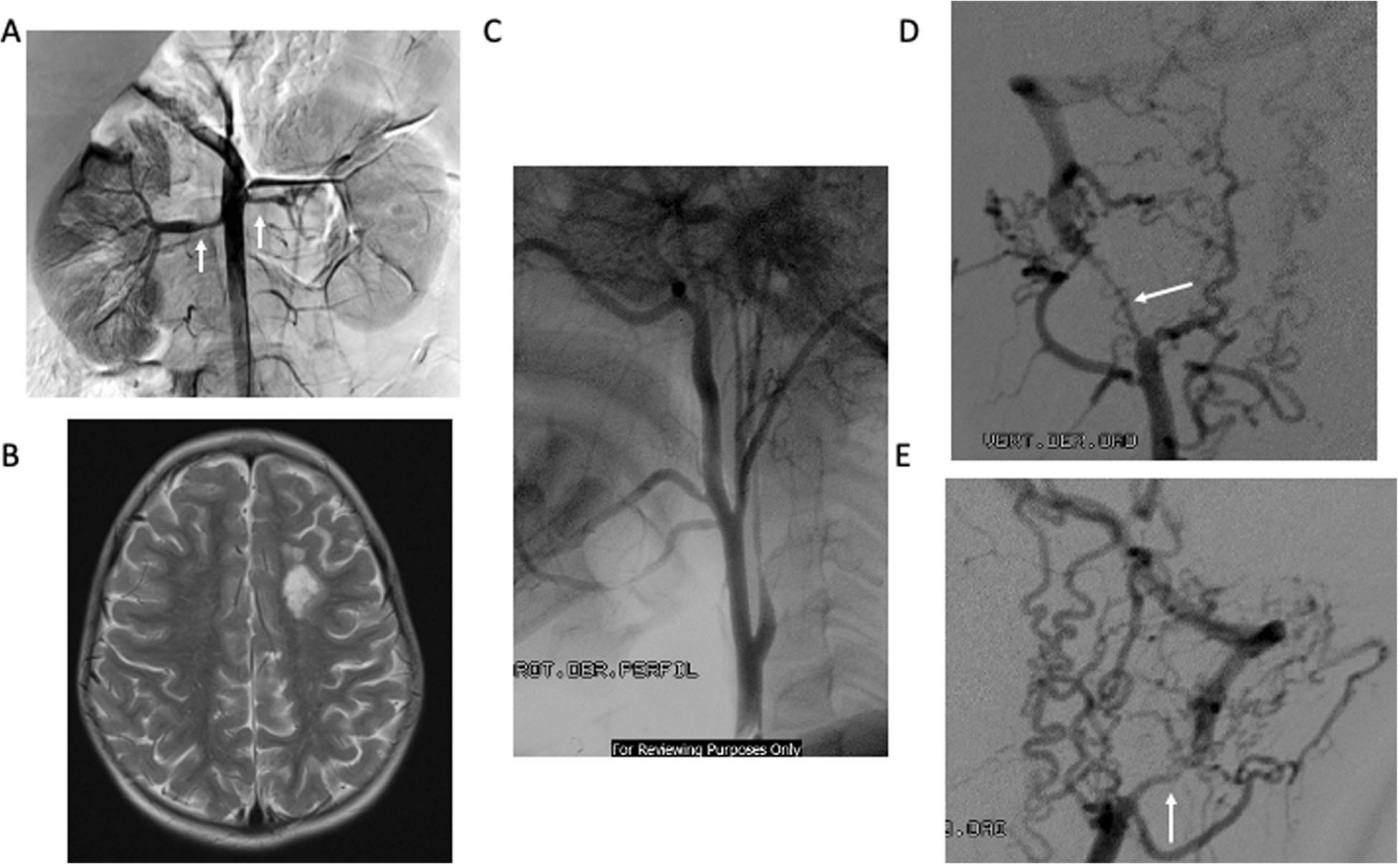
Scans results of patient. **a** Angiography image that shows bilateral renal artery stenosis (marked with arrows). **b** MRI—T2-weighted image showing left-hemisphere ischemic sequelae. **c** Angiography image showing right internal carotid stenosis. **d** Angiography image showing right vertebral artery stenosis. **e** Angiography image showing left vertebral artery stenosis

At the age of seven, she suffered a cerebrovascular accident. Magnetic resonance imaging showed ischemic sequelae in supraventricular white matter on the left hemisphere without evidence of bleeding (Fig. 1b). Doppler ultrasound showed severe stenosis of the right internal carotid (Fig. 1c). No areas of parietal enlargement or evidence of acute vasculitis were detected. Vertebral arteries were normal as well as left internal carotid. Angiography of neck vessels revealed severe stenosis of both vertebral arteries, severe stenosis in the right internal carotid (suprabulbar C1), and a short occlusion in segment C5 in the left internal carotid artery (Fig. 1d, e). Collateral circulation was present.

An echocardiogram showed minimal left ventricular hypertrophy with normal ejection fraction. Fundus examination was normal. No lactic acidemia, cerebrospinal fluid anomalies, nor thrombophilia elements, either hereditary or acquired, were detected. Treatment with AAS, clopidogrel, enalapril, and atorvastatin was started at this time.

Although she did not present Moya-Moya phenomenon, neurosurgeons decided to perform a bilateral indirect revascularization in 2 stages, left hemisphere at the age of 7 and right at the age of 8 years. No significant alterations were detected at artery wall in histopathologic analysis.

Renal circulation and causes of secondary hypertension were further studied: dosage of dopamine, adrenaline, noradrenaline, cortisol, adrenocorticotropic hormone, rennin, aldosterone, and vanillylmandelic acid were all normal. Renal scintigram (DMSA) was normal and both kidneys were equally functional. No evidence of cortical lesions was detected. At that time, Doppler ultrasound showed turbulent flux at the renal right artery, and angiography showed minimal artery wall irregularities but no significant stenosis. The left renal artery was normal.

At the age of 9 years, renovascular hypertension was diagnosed. Doppler ultrasound showed severe right renal artery stenosis and moderate stenosis of the left renal artery. Parenchymal circulation was normal in both kidneys. Renal angiography showed bilateral renal artery stenosis (> 50%); no areas of renal ischemia or aneurysms were detected (Fig. 1a). Given this diagnosis, enalapril was changed to propranolol. Urine examination was normal and renal function was conserved.

At the age of 10 years, Doppler ultrasound showed persistence of bilateral renal artery stenosis. Balloon angioplasty was performed: an incomplete dilatation was achieved, and no residual stenosis was detected at the time. No stent was placed. Renal function, ionogram, and blood gas analysis were normal after the procedure.

At the age of 11 years, stenosis of renal arteries reappeared: Doppler ultrasound showed stenosis (< 60%) and vascular wall irregularities in the right renal artery, and moderate-severe stenosis in the left renal artery (peak systolic velocity, 550 cm/s; increased renal-aortic ratio, 3:6). Parenchymal circulation was normal in both kidneys. Angio MRI will be performed (delayed due to the SARS-CoV-2 pandemic).

Surgical correction of hand syndactyly (left hand between the fourth and fifth finger) was previously done at the age of 1 year.

### Sequencing results

We did a whole genome sequencing (nuclear and mitochondrial DNA) on the patient with a target sequencing depth of 30×. We obtained 1,043,511,088 reads that passed QC-controls (according to samtools flagstat) and ~ 95% were mapped onto the reference genome (GRCh37). Variant calling analysis detected 4,893,483 variants that were further annotated and prioritized (see Variants filtering scheme).

For the mitochondrial genome, we obtained a high sequencing average depth of 7142× and a 100% coverage. A total of 401 variants were detected in the mtDNA.

### Frameshift mutation in trans with a large deletion in YY1AP1 gene is likely pathogenic

Two probable pathogenic variants were found in the gene *YY1AP1*, associated with the phenotype in question of recessive expression.

The first mutation is a single nucleotide deletion (YY1AP1:NM_001198903:exon10:c.1616delA:p.K539fs) causing a frameshift at the center of the gene (~ 60%, depending on the transcript). According to in silico predictions [10], the variant would not enter the nonsense-mediated decay (NMD) pathway but is nevertheless classified as “damaging.” This frameshift variant occurs at very low population frequency of 0.00003185, with no homozygous individuals being described in gnomad [11].

This frameshift was confirmed by Sanger sequencing in the patient and found in the mother in a heterozygous state (see the “Validation of relevant mutations in both parents and child” section).

**Fig. 2:**
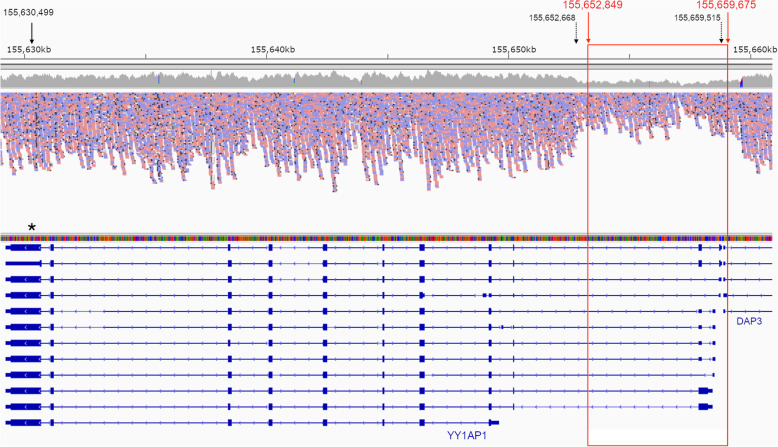
IGV view of the reads mapping onto the gene YY1AP1. At the bottom isoforms of the gene are shown. High blue boxes correspond to coding exons and lower boxes to non-coding regions. The asterisk shows the position of the frameshift variant. In broken (black) arrows, the position of the deletion, as estimated by the structural variant algorithm. In full (red) arrow, the corrected breakpoints’ positions as determined by Sanger sequencing

Additionally, upon visual inspection of YY1AP1’s read coverage, one potentially large intragenic deletion was detected, which comprehends the gene’s first coding exon (Fig. 2). Structural variant algorithm (see the “Variants filtering scheme” section, pipeline step 6) found one variant within the YY1AP1 gene, namely chr1:155652668-155659515 with a log2 copy ratio of − 0.97. The latter means that there are almost half (around −1) of the expected number of reads in that region, suggesting the presence of a large deletion. These findings were validated using a Sanger sequencing strategy (see the “Validation of relevant mutations in both parents and child” section), and following results were achieved:

i) Deletion was validated in child
ii) Deletion was found in the unaffected father and not in the mother
iii) Exact breakpoints are located in chr1:155.652.849–155.659.675 (Fig. 2)

Hence, we found a frameshift mutation in compound heterozygosity with a large deletion in the patient’s *YY1AP1* gene, being both parents’ healthy carriers.

### Expression data in publicly available databases shows expression of YY1AP1 isoforms affected by the deletion in relevant tissues

**Fig. 3:**
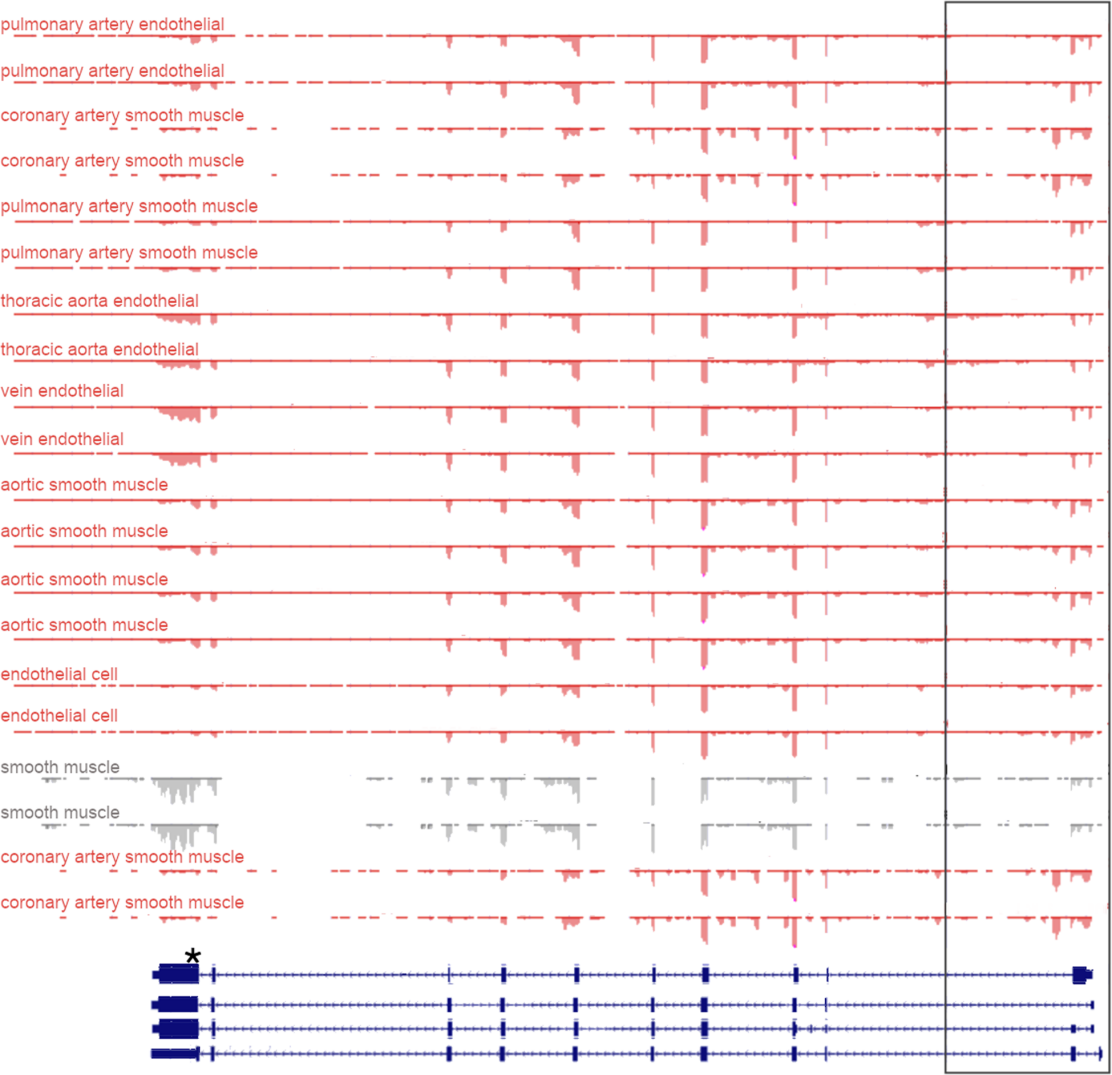
Expression of YY1AP1. RNA-seq expression profiles of *YY1AP1* in different endothelial and smooth muscle tissues. The box represents the deletion and the asterisk the frameshift variant. Data was downloaded from ENCODE

ENCODE database holds RNA-seq data of various “normal” tissues [12]. Of the available experiments, we included only total RNA-seq experiments (251). Out of the 57 available cell types, we considered the ones related to smooth muscle and endothelial cells, obtaining 21 experiments. We discarded uterine, trachea, bladder, and mammary microvascular, leaving 17 experiments for further analysis. Figure 3 shows a subset of 20 RNA-seq samples of those normal tissues. Reads are mapped onto the coding exons and shown as upside-down coverage bars (YY1AP1 gene is in the reverse strand). The corresponding isoforms are on the bottom (only 4 representatives that include different starting and final exons are represented). Expression signal is observed in the first exons showing that those isoforms are expressed at some level. Hence, those isoforms affected by the deletion are expressed in normal (relevant) tissues. Additionally, the last long exon (affected by the frameshift) is highly expressed in these tissues.

### Clinical findings concur with molecular diagnosis

According to the OMIM database, the *YY1AP1* gene is associated with Grange syndrome (ID 602531). Symptoms might include stenosis or occlusion of multiple arteries, including renal, abdominal, cerebral, and coronary arteries; congenital heart defects; brachydactyly; syndactyly; bone fragility; and learning disabilities.

The molecular diagnosis shed light on other clinical features that went previously unnoticed: hands and feet brachydactyly and mild cognitive deficit.

### Polygenic risk score for dyslipidemia

Since dyslipidemia is not a feature previously associated with YY1AP1 variants, we investigated other causes of dyslipidemia on a whole genome level, especially in genes associated with monogenic familial hypercholesterolemia (FH). The polygenic score calculated according to [13, 14] was above the threshold (> 0.73); hence, the patient was cataloged as polygenic hypercholesterolemia.

## Discussion

Here, we evaluated a patient with a rare disease via whole genome sequencing and found two novel variants that have the potential of being causative. According to ACMG variant interpretation guidelines [15], the frameshift variant corresponds to the PVS1 category (pathogenicity very strong), since it is a null variant, and it is located at a gene where the loss of function is a known mechanism of disease. It is also classified as PM2 due to the low frequency in databases (gnomad, 1000G). The deletion is also a null variant (exon deletion) and regarding the frequency, the same deletion is not observed in databases storing structural variants [16]; hence, it is classified as PM2. Additionally, for recessive disorders, the classification PM3 states that the variant should be detected in *trans* with a pathogenic variant. Since the frameshift and the deletion are in different chromosomes (maternal and paternal), both could be classified as PM3. According to ACMG rules, both variants are classified as pathogenic (1 PVS1 and 2 moderate pathogenics). Other pathogenic variants have been previously found in that gene’s vicinity (NM_001198903.1:c.1903_1906delTCTG; p.Glu636Profs) (https://www.ncbi.nlm.nih.gov/clinvar/variation/375641/), strongly suggesting that the entire genomic region is functionally relevant.

Moreover, we evaluated the probable protein product of the mutant (frameshift) YY1AP1 gene.

We cannot directly assess the fate of eventual truncated transcripts (NMD decay or other regulatory mechanisms) in the patient. This would require an extensive tissue culture project which is out of our scope. As for animal models, none seems readily available for this gene. For example, a protein product in *Mus musculus* (Uniprot accession E9Q507) harbors a domain 80% identical to ~ 220 N-terminal amino acids from YYAP1_HUMAN (one possible protein product). The mouse protein is a transcriptional co-regulator 2242 residues long, which makes it unsuitable for function transfer by homology or to understand the human protein with functional studies. Nevertheless, we may speculate on the protein product characteristics for the detected variants (e.g., there are no animal models available), but we may speculate on the protein product characteristics, were translation to occur for the frameshift variant. The most probable transcript would give a protein of 457 residues with two salient features: (i) several predicted disordered regions [17], with (ii) the highest disorder being predicted for the last 80 residues composing the C-terminal region. In contrast, the proteins produced by the wild-type gene are between 700 and 900 residues long, with the most disordered regions being flanked by N-terminal and C-terminal ordered ends. Thus, if transcription/translation occurred, we expect such protein products would have an aggregation tendency, hampering cellular function. The lack of suitable protein structure templates for both the wild-type and the mutant prevented us from gaining more precision on these variants’ molecular modeling and emit informed guesses on functional consequences. Nevertheless, the truncated protein might have self-aggregation tendencies because of this C-terminal disordered region, with numerous hydrophobic and positively charged residues. Furthermore, the missing ~ 350 residues are highly conserved in metazoans (in contrast to the N-ter region which is more variable), strongly suggesting selective functional pressures to preserve this ancient region.

Besides, we found some evidence to explain her dyslipidemia as a polygenic trait concurring with vascular lesions which are characteristic of *YY1AP1* LOF variants.

Additionally, another study has shown that immunoblot analysis of fibroblasts from a control and one affected Grange patient with two premature stop codon mutations showed the expected 88 kDa YY1AP1 protein in the control fibroblasts but no evidence of a full-length nor truncated protein in the proband’s fibroblasts [9]. The mutations present in that patient were p.Leu797*, which is predicted to produce an 80 kDa truncated protein with 91 residues deleted, and p.Gln242∗ for which a NMD mechanism was predicted. The frameshift variant found in the present patient was K539fs, which is predicted to produce a premature stop codon 12 positions downstream; therefore, it causes a truncation which is more upstream than the one in the position 797, and hence, it has probably an at least similar impact than the Leu797*. The large first-exons deletion we detected has probably a substantial impact on the resulting protein (e.g., NMD mechanism). So, the expected impact of our patients’ variants could be similar to that presented in the study above.

In conclusion, as an interdisciplinary group, we were able to diagnose a young girl with a rare disease that was very difficult to assess due to her highly heterogeneous symptoms. WGS revealed a compound heterozygosity in the *YY1AP1* gene. One of the variants was a large deletion that could have been missed by whole exome sequencing or other techniques, emphasizing its usefulness of this approach for complex cases.

## Conclusion

We think that this finding can contribute to the use of whole genome sequencing as a diagnostic tool of challenging rare diseases. Additionally, this kind of data might enhance the set of known mutations associated with different diseases. We think to share this information is crucial for pushing genomic medicine further and improve diagnostic yields.

## Materials and methods

### NGS sequencing and bioinformatics analysis

According to the manufacturer’s instructions, genomic DNA was extracted from 100 μl of whole blood using Qiamp® DNA Blood Mini kit (Qiagen, Germany).

We did a whole genome sequencing of the patient with 30X in a Hiseq X ten Illumina sequencer. The quality of reads was analyzed using FastQC [18], and reads were mapped onto the human genome (GRCh37) using BWA [19]. Variant calling was performed using GATK (according to best practices) [20]. ANNOVAR [21] was used then for the annotation. Different sets of filters were used in order to detect potentially causative mutations (see the “Variants filtering scheme” section).

The candidate frameshift mutation was further evaluated with the SIFT Indel tool [22], to estimate its pathogenicity effect.

Additionally, the mitochondrial genome was analyzed using MToolBox [23]. Structural variants were detected using BIC-seq2 [24, 25]

### Variants filtering scheme

In order to filter and prioritize the variants found, we used the following rationale:

1. Homozygous mutations in coding/splicing region with a population frequency lower than 1%
2. Heterozygous mutations in coding/splicing region with at least two variants in the same gene and a population frequency lower than 1% (compound heterozygous)
3. Heterozygous mutations in coding/splicing region with a population frequency less than 0.5%
4. Mitochondrial mutations with high heteroplasmy (> 10%) and in coding regions or tRNA or rRNA genes (and not part of the definition of the haplogroup and not in D-LOOP)
5. Non-coding variants, either with “uncertain significance” (VUS) or “pathogenic/Likely pathogenic” or “conflicting interpretations of pathogenicity” classifications, as determined by ClinVar [26]
6. Structural variants in potentially relevant genes/chromosomes

### Validation of relevant mutations in both parents and child

Sanger sequencing was performed to confirm the patient’s frameshift variant and validate it in the parents. Regarding the large deletion, the first step was to confirm it in the patient and check whether it was present in either parent. For this purpose, we performed a PCR amplification, using primers on each side of the potential deletion. In this case, breaking points as estimated by NGS were considered. The expected PCR product in wild-type individuals is 7251 and for individuals carrying the deletion ~ 404 (Table S2). Figure S1 A shows PCR products in a gel, confirming the presence of the patient’s mutation (ER13) in the father (F). Mother (M) shows no copy of the deletion. The next step was to determine the exact breaking points’ positions of the deletion. PCR product in Figure S1 B shows the primer design for Sanger sequencing and PCR amplification (same set). Here, individuals carrying the mutation were sequenced with Sanger to validate the breaking point and the primers were designed on each side of the estimated deletion.

To analyze the zygosity of the large deletion in the patient and parents more in detail, we performed several PCRs with different pairs of primers. First, a set of primers spanning the 5′ breakpoint (P_F_5: primer forward 5′ and P_R_5: primer reversed 5′) was designed in order to amplify an amplicon of ~ 551 bases in a wild-type genome. The second set of primers was designed to amplify the 3′ breakpoint (P_F_3 and P_R_3). The resulting amplicon would be ~ 482 bases long in a wild-type genome. Using this design, an individual not carrying the large deletion in either allele would show two bands in an agarose gel, one of length 551 and the other 482 (figure S1 D). On the contrary, an individual carrying the large deletion in a heterozygous state, would show three sets of amplicons: the two amplicons defined above (P_F_5-P_R_5 and P_F_3-P_R_3) and one additional amplicon, constructed from P_F_5 and P_R_3 (figure S1 C, D). This amplicon should have a length of ~ 428 (318 bp from P_F_5 to the first breakpoint and 110 bp from the second breakpoint to P_R_3). We found that the mother has two wild-type alleles and the father and patient have both one copy of the deleted allele.

### Polygenic risk score calculation

The genetic risk assessment was completed by calculating a polygenic risk score for FH according to [13, 14]. Using the WGS, we assess the SNPs in Table S1 and calculated the score for the patient.

## Supplementary Information

**Additional file 1: Table S1.** Polygenic risk score. SNPs considered for the hypercholesterolemia familial polygenic risk score. **Table S2.** Primers used for amplification of the large deletion.

**Additional file 2: Figure S1.** Confirmation of deletion and frame-shift variant in patient and parents.

## Data Availability

The datasets used and/or analyzed during the current study are available from the corresponding author on reasonable request.

## References

[1] European Commission – European Commission (2020). Rare diseases.

[2] Clark MM, Stark Z, Farnaes L, Tan TY, White SM, Dimmock D, Kingsmore SF (2018). Meta-analysis of the diagnostic and clinical utility of genome and exome sequencing and chromosomal microarray in children with suspected genetic diseases. NPJ Genom Med.

[3] Mercimek-Mahmutoglu S, Patel J, Cordeiro D, Hewson S, Callen D, Donner EJ, Hahn CD, Kannu P, Kobayashi J, Minassian BA, Moharir M, Siriwardena K, Weiss SK, Weksberg R, Snead OC (2015). Diagnostic yield of genetic testing in epileptic encephalopathy in childhood. Epilepsia.

[4] Della Mina E, Ciccone R, Brustia F, Bayindir B, Limongelli I, Vetro A (2015). Improving molecular diagnosis in epilepsy by a dedicated high-throughput sequencing platform. Eur J Hum Genet.

[5] Liu HY, Zhou L, Zheng MY, Huang J, Wan S, Zhu A, Zhang M, Dong A, Hou L, Li J, Xu H, Lu B, Lu W, Liu P, Lu Y (2019). Diagnostic and clinical utility of whole genome sequencing in a cohort of undiagnosed Chinese families with rare diseases. Scientific Reports.

[6] Grange DK, Balfour IC, Chen S, Wood EG (1998). Familial syndrome of progressive arterial occlusive disease consistent with fibromuscular dysplasia, hypertension, congenital cardiac defects, bone fragility, brachydactyly, and learning disabilities. Am J Med Genet.

[7] Weymann S, Yonekawa Y, Khan N, Martin E, Heppner FL, Schinzel A, Kotzot D (2001). Arterial occlusive disorder and brachysyndactyly in a boy: a further case of Grange syndrome?. Am J Med Genet.

[8] Wallerstein R, Augustyn AM, Wallerstein D, Elton L, Tejeiro B, Johnson V, Lieberman K (2006). New case of Grange syndrome without cardiac findings. Am. J. Med. Genet..

[9] Guo D, Duan XY, Regalado ES, Mellor-Crummey L, Kwartler CS, Kim D, Lieberman K, de Vries BBA, Pfundt R, Schinzel A, Kotzot D, Shen X, Yang ML, Bamshad MJ, Nickerson DA, Gornik HL, Ganesh SK, Braverman AC, Grange DK, Milewicz DM, University of Washington Center for Mendelian Genomics (2017). Loss-of-function mutations in YY1AP1 lead to Grange syndrome and a fibromuscular dysplasia-like vascular disease. Am. J. Hum. Genet..

[10] SIFT missense predictions for genomes. Nat Protocols. 2016;11(1):1–9. 10.1038/nprot.2015.123. 10.1038/nprot.2015.123 26633127

[11] Karczewski KJ, Francioli LC, Tiao G (2020). The mutational constraint spectrum quantified from variation in 141,456 humans. Nature.

[12] Davis CA, Hitz BC, Sloan CA, Chan ET, Davidson JM, Gabdank I, Hilton JA, Jain K, Baymuradov UK, Narayanan AK, Onate KC, Graham K, Miyasato SR, Dreszer TR, Strattan JS, Jolanki O, Tanaka FY, Cherry JM (2018). The Encyclopedia of DNA elements (ENCODE): data portal update. Nucleic Acid Res.

[13] Talmud PJ, Shah S, Whittall R, Futema M, Howard P, Cooper JA, Harrison SC, Li KW, Drenos F, Karpe F, Neil HAW, Descamps OS, Langenberg C, Lench N, Kivimaki M, Whittaker J, Hingorani AD, Kumari M, Humphries SE (2013). Use of low-density lipoprotein cholesterol gene score to distinguish patients with polygenic and monogenic familial hypercholesterolaemia: a case-control study. The Lancet.

[14] Futema M, Bourbon M, Williams M, Humphries SE (2018). Clinical utility of the polygenic LDL-C SNP score in familial hypercholesterolemia.

[15] Richards S, Aziz N, Bale S (2015). Standards and guidelines for the interpretation of sequence variants: a joint consensus recommendation of the American College of Medical Genetics and Genomics and the Association for Molecular Pathology. Genet Med.

[16] Lappalainen I, Lopez J, Skipper L, Hefferon T, Spalding JD, Garner J, Chen C, Maguire M, Corbett M, Zhou G, Paschall J, Ananiev V, Flicek P, Church DM (2013). DbVar and DGVa: public archives for genomic structural variation. Nucleic Acids Res..

[17] Buchan DWA, Jones DT (2019). The PSIPRED Protein Analysis Workbench: 20 years on. Nucleic Acids Res.

[18] Andrews S (2010). FastQC: a quality control tool for high throughput sequence data.

[19] Li H, Durbin R (2009). Fast and accurate short read alignment with burrows-wheeler transform. Bioinformatics.

[20] Mckenna A, Hanna M, Banks E, Sivachenko A, Cibulskis K, Kernytsky A (2010). The genome analysis toolkit: A mapreduce framework for analyzing next-generation dna sequencing data. Genome Research.

[21] Wang K, Li M, Hakonarson H. Annovar: functional annotation of genetic variants from high-throughput sequencing data. Nucleic Acids Res. 2010;38(16):e164. 10.1093/nar/gkq603. Epub 2010 Jul 3. 10.1093/nar/gkq603 PMC2938201 20601685

[22] Ng PC, Henikoff S (2003). SIFT: Predicting amino acid changes that affect protein function. Nucleic Acids Res..

[23] Calabrese C, Simone D, Diroma MA, Santorsola M, Gutta C, Gasparre G (2014). MToolBox: A highly automated pipeline for heteroplasmy annotation and prioritization analysis of human mitochondrial variants in high-throughput sequencing. Bioinformatics.

[24] Xi R, Lee S, Xia Y, Kim T, Park P (2016). Copy number analysis of whole-genome data using BIC-seq2 and its application to detection of cancer susceptibility variants. Nucleic Acids Res.

[25] Xi R, Hadjipanayis AG, Luquette LJ, Kim TM, Lee E, Zhang JH, Johnson MD, Muzny DM, Wheeler DA, Gibbs RA, Kucherlapati R, Park PJ (2011). Copy number alteration detection in sequencing data using the Bayesian information criterion. Proceed Nat Acad Sci.

[26] Landrum MJ, Lee JM, Benson M, Brown GR, Chao C, Chitipiralla S, Gu B, Hart J, Hoffman D, Jang W, Karapetyan K, Katz K, Liu C, Maddipatla Z, Malheiro A, McDaniel K, Ovetsky M, Riley G, Zhou G, Holmes JB, Kattman BL, Maglott DR (2018). ClinVar: improving access to variant interpretations and supporting evidence. Nucleic Acids Res..

