# Supplementary material for: Whole genome sequencing reveals a frameshift mutation and a large deletion in YY1AP1 in a girl with a panvascular artery disease
Source: Hum Genomics. 2021 May 10;15:28. doi: 10.1186/s40246-021-00328-1 (PMC8108437; doi:10.1186/s40246-021-00328-1)
Supplement: Supplementary file 1 — Additional file 1: Table S1. Polygenic risk score. SNPs considered for the hypercholesterolemia familial polygenic risk score. Table S2. Primers used for amplification of the large deletion. [file 40246_2021_328_MOESM1_ESM.pdf]

Supplementary Table S1: SNPs considered for the hypercholesterolemia familial polygenic risk score

rsid	ref allele	alt allele	genotype	
rs2479409	G	A	0/1	
rs629301	G	T	0/1	
rs1367117	G	A	0/1	
rs4299376	G	T	0/1	
rs1564348	T	C	0/1	
rs1800562	G	A	0/0	
rs3757354	C	T	0/0	
rs11220462	G	A	0/0	
rs8017377	G	A	0/0	
rs6511720	G	T	0/0	
APOE			e3/e3	
rs429358	T	C	0/0	
rs7412	C	T	0/0	

Supplementary Table S2: Primers used for amplification of the large deletion

Primer ID	Sequence (5'->3')	Length	Strand	Amplicon Size	Description
1:155652533-155652556	ACAGGCACACAGTGTCTCTTCCTC	24	Forward	WT copy : 7251-bp<br>Del copy : 404-bp	Primer for Long Amplification (Fwd)
1:155659784-155659762	ACGGCAATACTCTGCACCTTCCC	23	Reverse		Primer for Long Amplification (Rev)
1:155652533-155652556 (PF5)	ACAGGCACACAGTGTCTCTTCCTC	24	Forward	WT copy: 551-bp<br>Del copy : -	Primer for Long Amplification (Fwd)
1:155653083-155653061 (PR5)	TATGTGCCAAGCACTATTCCAGG	23	Reverse		Primer that amplify the sequence spanning 5' breakpoint
1:155659303-155659326 (PF3)	GGACTCAGTTTCCCTTGTCTTCAG	24	Forward	WT copy : 482-bp<br>Del copy : -	Primer that amplify the sequence spanning 3' breakpoint
1:155659784-155659762 (PR3)	ACGGCAATACTCTGCACCTTCCC	23	Reverse		Primer for Long Amplification (Rev)

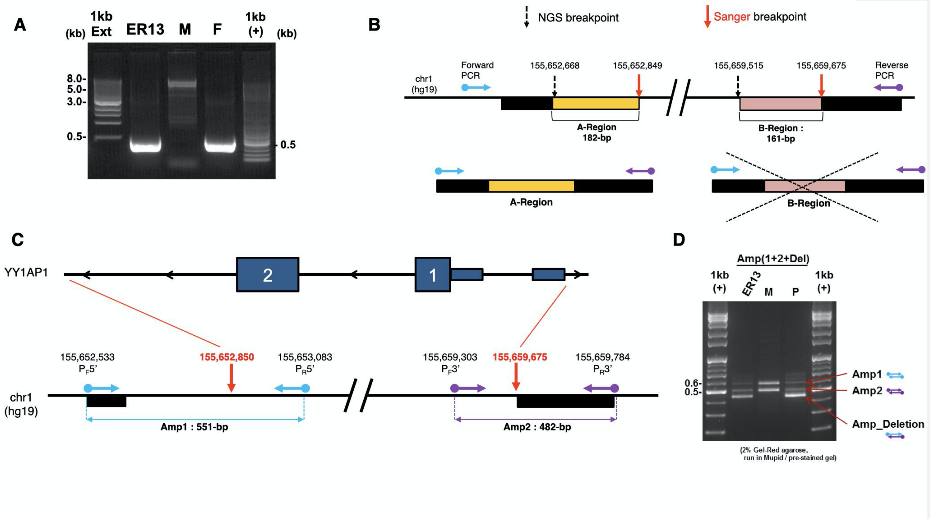

Supplementary Figure S1. Confirmation of deletion and frame-shift variant in patient and parents.

Eliminado: S3

A: PCR products in gel. Primers were designed according to the NGS breaking point estimations. B: Predicted PCR product for individuals carrying the mutation. Only A-region would be observed (black-yellow-black). B-region would not be observed. Wild-type individuals would show A and B-regions. C: Primer design to evaluate zygosity. Amp1 and Amp2 are present in wild-type genomes. Amp determined by PF5 and PR3 (Amp_deletion) is only present in the individuals with the deletion. D: PCR products in gel. Father and patient (ER13) have all three aplicons. Mother has only two wild-types.
