# Supplementary figures and images for: Whole genome sequencing reveals a frameshift mutation and a large deletion in YY1AP1 in a girl with a panvascular artery disease
Source: Hum Genomics. 2021 May 10;15:28. doi: 10.1186/s40246-021-00328-1 (PMC8108437; doi:10.1186/s40246-021-00328-1)

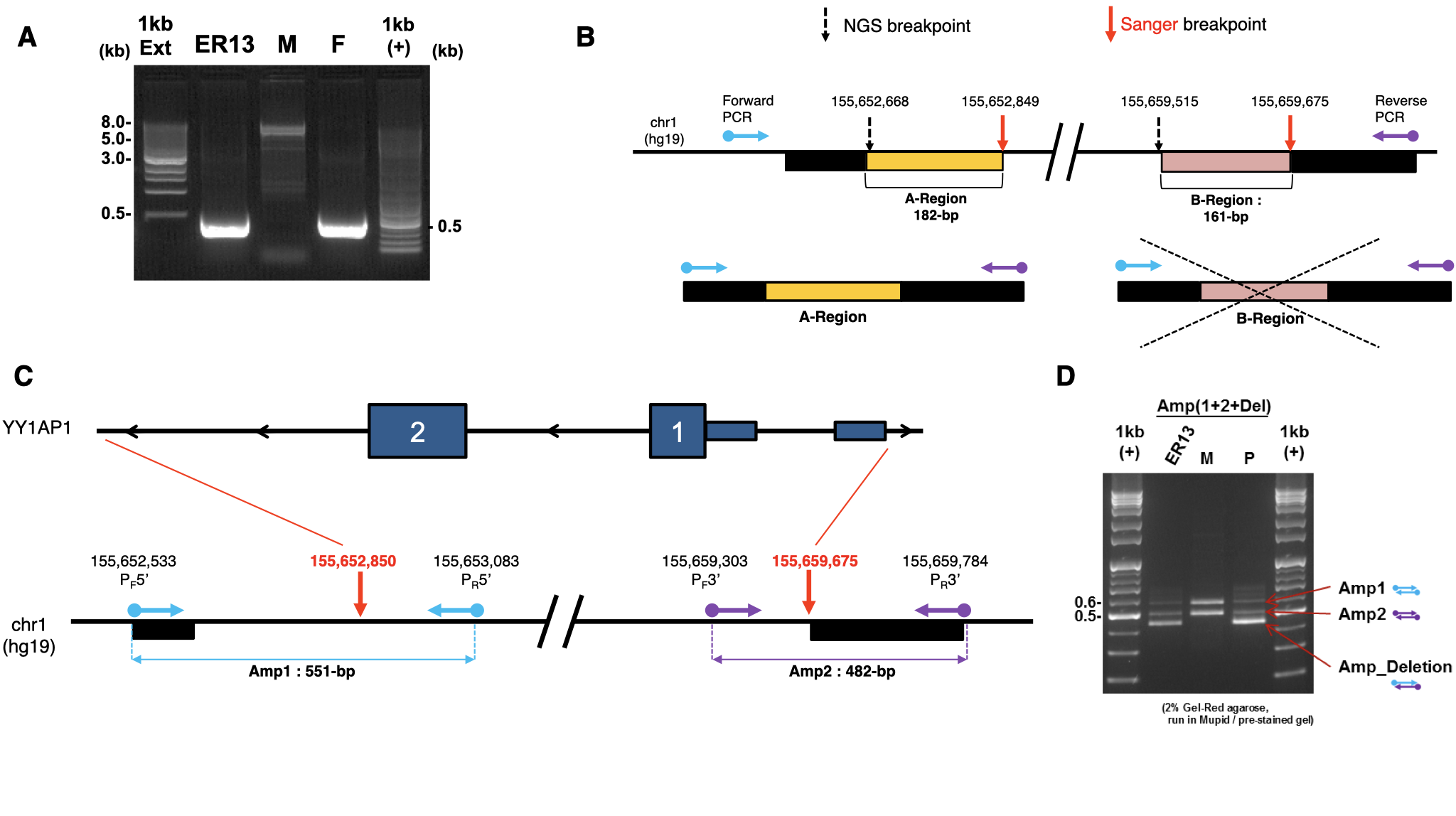

Supplement: Supplementary file 2 — Additional file 2: Figure S1. Confirmation of deletion and frame-shift variant in patient and parents. [file 40246_2021_328_MOESM2_ESM.tiff]
